# Malaria and malnutrition in children under the Seasonal Malaria Chemoprevention (SMC) coverage in the health district of Nanoro, Burkina Faso

**DOI:** 10.1371/journal.pone.0342237

**Published:** 2026-02-23

**Authors:** Eulalie Wendingouda Compaore, Paul Sondo, Bérenger Kabore, Toussaint Rouamba, Amélé Fifi Chantal Kouevi, Ipéné Mylène Carenne Bayala, Sié A. Elisée Kambou, Kié Solange Millogo, Ismaïla Bouda, Delwendé Florence Ouedraogo, So-vii Franck Hien, Adama Kazienga, Karim Derra, Eli Rouamba, Awa Gneme, Halidou Tinto

**Affiliations:** 1 Institut de Recherche en Sciences de la Santé/ Clinical Research Unit of Nanoro (IRSS-URCN), Nanoro, Burkina Faso; 2 Laboratory of Animal Biology and Ecology (LBEA)/ UFR SVT/ Université Joseph Ki Zerbo, Ouagadougou, Burkina Faso; Para Federal University, BRAZIL

## Abstract

**Introduction:**

Malaria and malnutrition are major causes of childhood morbidity and mortality. Most of children receiving Seasonal malaria chemoprevention (SMC) are also generally malnourished during SMC delivery period. This study aimed at assessing the risk of malaria infection in malnourished children, compared to children with an adequate nutritional status (ANS) during the delivery period of SMC intervention in the health district of Nanoro, Burkina Faso.

**Methods:**

A longitudinal survey of children aged 6–59 months receiving SMC intervention was carried out between 2020 and 2022 in Nanoro, Burkina Faso. WHO online anthro survey tool was used to assess the nutritional status. Included children were stratified into two sub-groups as malnourished from the start of the first SMC round until one month after the last SMC round and those of ANS during that same period. In addition to the monthly home visits, all health care attendance were recorded for all participants. At each visit, socio-anthropometric data were collected. Blood smears were collected for malaria diagnosis by rapid diagnostic test (RDT) and microscopy. The effect of the nutritional status on the occurrence of uncomplicated malaria was assessed using a negative binomial model, with results expressed as incidence risk ratio (IRR).

**Results:**

Out of the 425 children included this study, 260 were malnourished, while 165 children had an ANS. Malaria incidence per 1000 child-month were higher in undernutrition (974.47) than in ANS (214.47). Malnourished children were 1.41 times more likely to have t an episode of clinical malaria than children with ANS (IRR 1.42; 95% CI 1.03–1.94; p = 0.028).

**Conclusion:**

There was an increased risk of malaria infection in undernourished children compared to children with an adequate nutritional status suggesting the need of combining SMC intervention with nutrients supplementation to achieve best impact for malaria control in food insecure areas.

## Introduction

Malaria remains a major public health problem worldwide, with an estimated 269 million cases and 597,000 deaths in 2023 [1]. Africa alone accounted for 94% of malaria cases and 95% of malaria related deaths with children under 5 years t of age bearing the heaviest burden, accounting for up to 76% of these deaths [1]. In Burkina Faso, malaria remains a major health challenge with 9,967,712 cases and 3,385 malaria related deaths recorded in 2023. under5 years of age being the most affected with 3,380,291 cases and 2,216 deaths in 2023 [2]. To tackle this devastating disease, Burkina Faso has implemented several control strategies as per the world health organisation (WHO) recommendation, including the seasonal malaria chemoprevention(SMC) [3]. SMC is a large-scale community-based intervention consisting in a monthly administration of a complete dose of Amodiaquine (AQ)+Sulfadoxine-Pyrimethamine (SP) during the high transmission season, to children aged 3–59 months [3,4]. SMC is a reliable intervention with promising results at evaluation phase leading up to 76% reduction of the incidence of uncomplicated malaria and by 42% the malaria-related mortality [5].

In Burkina Faso, SMC is being implemented since 2014 and was scaled across the entire country in 2018 with a high coverage rate [6,7]. However, malaria burden remains higher among children under SMC coverage in Burkina Faso. This results from multiples factors including non-compliance to other preventives measures such as the use of long-lasting insecticide nets (LLINs), the emergence and spread of resistant parasites to AQSP (Amodiaquine and Sulfadoxine-Pyrimethamine) [8,9], the lack of compliance with the second and third doses of Amodiaquine by children’s parents or legal guardians [10], and the higher prevalence of asymptomatic carriers among other members of the households surrounding children under SMC coverage [11]. In such a context, there is a crucial need for new initiatives tailored to local context in order to boost the impact of the SMC intervention [2].Indeed, undernutrition is also a public health problem in sub-Saharan Africa, affecting children’s growth, immunity and resistance to infection [12–15]. In Burkina Faso, malnutrition (like malaria) represents a serious threat, affecting children under five years old especially during the rainy season coinciding with a period of food shortage in the country. In 2023, the prevalence of malnutrition in Burkina Faso was estimated at 30.6% and the Centre-West region including the health district of Nanoro is one of the areas with the highest prevalence in the country [2].The prevalence of acute malnutrition was 7.25%, underweight rate was 23.38% and stunted growth was 48.79% in children under 5 years of age [16]. Besides the negative effect on children’s immune systems with increasing risk of infections, including malaria [17,18], malnutrition has direct consequences on treatment efficacy through a poor absorption of medicines leading to under-dosing of treatments in these children [19]. For instance, severely undernourished children were at risk of underdosing of lumefantrine, resulting in an increased risk of malaria re-infection [20]. All these aspects support the hypothesis that malnutrition could be a factor that could negatively affect the effectiveness of the SMC intervention. Though children with severe acute malnutrition are not eligible to SMC, the majority of children receiving SMC are moderately undernourished during SMC delivery period in Burkina Faso [16] yet the effect of nutritional status on the response to SMC intervention remain poorly assessed. This study aimed at assessing the risk of malaria infection as response to SMC in malnourished children, compared to children with an adequate nutritional status (ANS) during the SMC delivery period in the health district of Nanoro, Burkina Faso.

## Materials and methods

### Study area

The study was carried out in the health district of Nanoro, located in the Center-Western region in Burkina Faso. The Nanoro Health District (DSN) covers an area of 1,302 km^2^, accounting for 5.98% of the 21,752 km^2^ of the Center-West region of Burkina Faso [21]. The area is characterised by a Sudano-Sahelian climate with a long dry season from November to May and a short rainy season from June to October. The average rainfall is estimated at between 450 and 700 mm per year [21].Malaria is endemic and more than 126,294 malaria cases were reported in 2023 in the health district of Nanoro [2]. Malaria transmission is seasonal, with high transmission during the rainy season and low transmission during the dry season [22–24], justifying the suitability of the area for SMC intervention. Data collection was performed in the catchment area of five peripheral health centers of the commune of Soaw (Soaw, Zoetgomdé, Kolokom, Poéssé) and one of the commune of Nanoro (Kokolo). The total population of the study catchment area was estimated at 193,777 in 2024 [21].

### Study design

This was a longitudinal survey carried out between 2020 and 2022 in children aged 6–59 months receiving SMC intervention. The study was embedded to two major projects aiming at improving the impact of SMC intervention in the health district of Nanoro, Burkina Faso. Details of the two projects SMC-NUT (NCT04238845) and SMC-RST (NCT04816461) were previously described elsewhere [25,26]. Recruitments occurred between July 07, 2020 and July 30, 2020 for SMC-NUT and between August 04, 2021 and July 07, 2022 for SMC-RST.

However, for this survey, only children of the control (no intervention) arms of the two projects were considered for the investigation.SMC was administered at each monthly visit, from July to October, by the Nanoro health district, in accordance with the guidelines of the Permanent Secretariat for the Elimination of Malaria (SP/Palu) [27]. At each visit, the research team ensured that the child had received the anti-malarial treatment before collecting socio-anthropometric data and blood samples. These samples were used to measure haemoglobin levels, carry out rapid diagnostic tests (RDTs), as well as thick blood drops and blood smears (GE/FS). Parents or legal guardians were asked to go to their usual health centre if their child developed a fever during the implementation of the SMC. If malaria was diagnosed, treatment was immediately administered to the child, in accordance with the recommendations of the World Health Organisation [28].

### Data collection

At enrolment and during the monthly home visits or at any attendance at heath facilities, weight, height and mid-upper arm circumference (MUAC) were measured. Weight was measured using a SECA 806 scale while height was measured using a SECA 206 tape measure, attached to a specially designed 2.20 metres wooden stand, and recorded in centimetres. MUAC was measured halfway between the point of the shoulder and the point of the elbow using a Shakir strip.

Haemoglobin was measured using a HemoCue® 801 + analyser (SOC-HE121916, Danayer group, Angelholm, Sweden). A non-contact clinical thermometer (Microlife NC 200, Switzerland) was used to measure the body temperature. The temperature was taken at a distance of 1–5 cm from the forehead and the value was automatically read in degrees Celsius.

### Malaria diagnosis

SD Bioline (SD Standard Diagnostics, INC., Republic of Korea) histidine-rich protein 2 (HRP2) RDTs, were used for rapid detection of malaria infection. Thereafter, thick drop and blood smear stained with 3% Giemsa for 30 minutes were made and were double read using an Olympus CX21 microscope (Olympus Corporation, Tokyo, Japan). Parasite density was determined by counting the number of asexual parasites per 200 white blood cells and calculated per microlitre of blood, assuming a white blood cell count of 8000/μl. Slides were declared negative when no asexual parasites were observed after the reading at least 100 microscopic fields [29].

### Data management and Statistical Analysis

Included children were stratified into two sub-groups either malnourished or ANS. Only children who kept similar nutritional status from the start of the first SMC round until one month after the last SMC round were considered in this study, excluding children who shifted from malnourished to ANS or vice versa. Furthermore, children who missed at least two consecutive home visits over the five visits during the SMC period were also excluded from the analysis. Nutritional status for each participant was estimated on a monthly basis over the study period using the WHO Anthro survey online tool (WHO Anthro Survey Analyser) [30]. Using this tool and providing the input parameters (Age, sex, weight, and height) resulted in an estimated value of nutritional status indicators such as weight for age Z-score (WAZ), height for age Z-score (HAZ), and weight for height Z-score (WHZ) for each participant and classified them into stunting, wasting, and underweight respectively using the cut-off of less than −2 Z score (<−2 Z score). Undernutrition status was defined as participants having at least one of these indicators (stunting, wasting, and underweight), while adequate participants were those without any of these signs.

To ensure the stability of the nutritional status classification, only children who had at least three consecutive visits and maintained the same nutritional status across those visits were included in the analysis. To account for the differences in observation time in the modelling, person-time in months was calculated for each child, corresponding to the total number of months under observation.

Descriptive statistics were performed using the proportion and geometric mean (95% confidence interval) for qualitative variables and parasite density. Mean (standard deviation) or median (Q1 - Q3) were used for continuous variables for standard and non-normal distributed data, respectively. A bar plot of the crude malaria incidence in child-month per 1000 was also presented for each nutritional status separately (adequate nutritional status and undernutrition). We also assessed whether RDT positivity differed between nutritional status over time using a mixed-effects logistic regression model. To this end, the binary outcone was the monthly RDT result (positive/negative) recorded for each participant across five visits. To account for within-individual correlation, we specified random intercept and slopes for time in months at the individual level. The key parameter of interest was the interaction between the time (in month) and the nutritional status, which tested whether the trajectory of RDT positivity over time differed between nutritional status.

Furthermore, the effect of the nutritional status and other potential predictors (age, gender, and study) on the occurrence of uncomplicated malaria was assessed by considering the number of malaria episodes over the study period as the main outcome and incorporating the natural logarithm of person-time as an offset. To this end, a negative binomial model, with results expressed as incidence risk ratio (IRR) was used. A univariate analysis was performed, and variables with a p-value < 0.20 were included in multivariable analyses.

Data were analyzed using R version 4.3, and Stata version 14 (StataCorp, College Station, Texas, USA), and a p-value less than 0.05 was considered statistically significant.

### Ethical consideration

The two studies (SMC-NUT and SMC-RST) were conducted in compliance with fundamental ethical principles and applicable national, European and international regulations. They have been approved by the Burkina Faso Health Research Ethics Committee under numbers: No. 2020-01-007 of 01/05/2020 for SMC-NUT and No. 2021-03-059 of 10/03/2021 for SMC-RST. Written informed consent was obtained from the children’s parents or legal guardians before enrolment in the study.

## Results

A total of 884 children (353 from SMC-NUT study and 531 from SMC-RST study) were eligible for this investigation. Out of them, 76 children missed at least two consecutive visits while a change of the nutritional status over the follow-up period was observed in 383 children.

The baseline characteristics of the study population are presented in Table 1. Undernutrition was highly prevalent among children under SMC coverage, accounting for 61% (260/425), *versus* 39% (165/425) with an ANS. Children aged 2 years or over were mostly represented in the two sub-groups accounting for 71.54% (186/260) of the undernourished children and 86.67% (143/165) of children with an ANS. Gender was almost equally balanced in both groups. The mean weight was 13.76 kg (SD: 2.19) in children with ANS versus 10.51 kg (SD: 2.52) in undernourished children. Similarly, the median height of children with ANS were higher than those of undernourished children, 94 cm (Q1 - Q25: 90–100) versus 88 cm (Q1 - Q25: 78–95). Mean temperature, mean MUAC and mean haemoglobin were almost similar in the two groups of children. The geometric mean parasite density observed was slightly higher in the malnourished children 868P/μl (95%CI: 458–1645) than in those with an ANS 734P/μl (95% CI: 339–1587).

**Table 1:** Baseline socio-demographic characteristics.

Variable	Overall	Nutritional status	
		Undernutrition	Adequate nutritional status
**Age group (years)**			
**n**	425	260	165
**< 2**	96 (22.59)	74 (28.46)	22 (13.33)
**≥ 2**	329 (77.41)	186 (71.54)	143 (86.67)
**Gender**			
**n**	425	260	165
**Male**	221 (52.00)	144 (55.38)	77 (46.67)
**Female**	204 (48.00)	116 (44.62)	88 (53.33)
**Weight (kg)**			
**n**	424	259	165
**Mean (SD)**	11.77 (2.87)	10.51 (2.52)	13.76 (2.19)
**Height (cm)**			
**n**	425	260	165
**Median(Q1 – Q75)**	88 (78–95)	80 (72–90)	94 (90–100)
**Temperature (C)**			
**n**	423	258	165
**Mean (SD)**	36.24 (0.56)	36.24 (0.59)	36.23 (0.52)
**MUAC (cm)**			
**n**	422	258	164
**Mean (SD)**	14.70 (1.22)	14.28 (1.17)	15.36 (1.00)
**Haemoglobin (g/dL)**			
**n**	388	237	151
**Mean (SD)**	10.09 (1.40)	9.89 (1.47)	10.41 (1.21)
**Parasitemia (P/μl)**			
**n**	67	46	21
**GMPD**^**#**^**(95%CI)**	824 (505–1345)	868 (458–1645)	734 (339–1587)

^#^GMPD: geometric mean parasite density.

^#^SD: Standard deviation

^#^CI: confidence interval.

### Effect of the nutritional status of children on the incidence of clinicalmalariaduring the SMC delivery period

**Fig 1:**
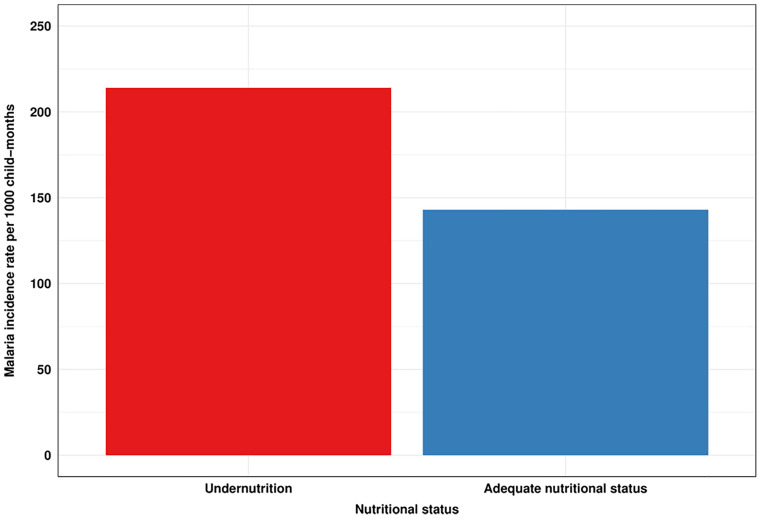
Malaria incidence rate according to nutritional status. This figure illustrates the crude malaria incidence rate for children with an ANS and malnourished children. To estimate these incidence rates, the number of episodes and the total time at risk in child-month for each nutritional status were first calculated. Next, the incidence rate was calculated by dividing the number of clinical episodes by the total time at risk. The blue colour represents children with an ANS, while the red indicates undernourished children.

During the follow-up, 299 episodes of clinical malaria were recorded in the total population. According to nutritional status, the number of malaria episodes was significantly higher in malnourished children (n = 209) than in children with ANS (n = 90). The total time at risk in persons-month and the malaria incidence per 1000 child-month were respectively higher in undernutrition (974.47 and 214.47) than in ANS (627.89 and 143.337). Fig 1 shows the cumulative episodes of uncomplicated malaria as a function of nutritional status over the study period (Fig 1).

Children’s age and nutritional status were significantly associated with the risk of contracting clinical malaria while no effect of gender was observed. Children aged two years and over had a higher risk of contracting uncomplicated malaria than those under two (IRR 0.64; 95% CI 0.47–0.87; p < 0.005). In addition, malnourished children were 1.41 times more likely to present an episode of uncomplicated malaria than those with ANS (IRR 1.42; 95% CI 1.03–1.94; p = 0.028). The effect of nutritional status on the incidence of uncomplicated malaria are presented in Table 2.

**Table 2:** Effect of nutritional status on the incidence of uncomplicated malaria.

Characteristic	Risk factor	Unadjusted		Adjusted	
		IRR (95%CI)	p-value	IRR (95%CI)	p-value
**Age (in years)**	< 2	1		1	
	≥ 2	0.61 (0.45–0.82)	0.001	0.64 (0.47–0.87)	0.005
**Sex**	Female	1			
	Male	1.01 (0.76–1.35)	0.903		
**Nutritional status**	Adequate nutritional status	1	–	1	–
	Undernutrition	1.49 (1.09–2.05)	0.012	1.42 (1.03–1.94)	0.028

### Effect of nutritional status on RDT positivity rate during the monthly home visits

**Fig 2:**
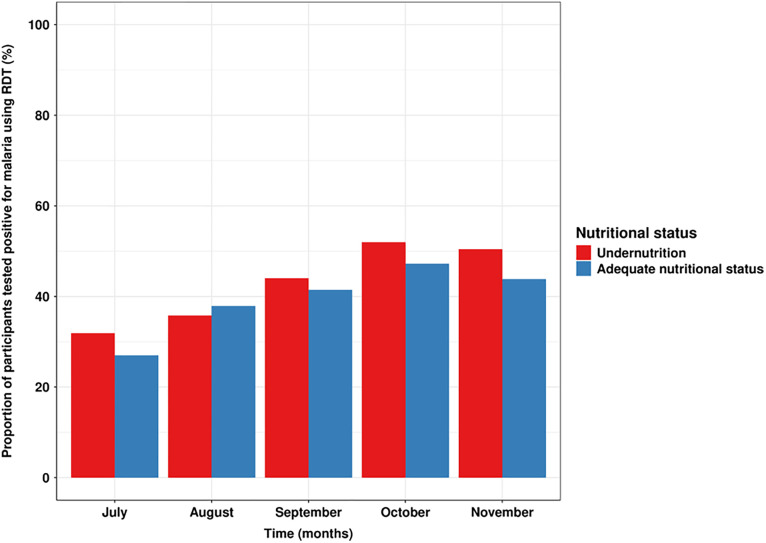
Cross-sectional RDT positivity rate per nutritional status over SMC period. This figure shows the proportion of children tested positive for malaria when deploying the RDT. To this end, monthly visits data were used to estimate the proportions of infected children to malaria when using RDT per nutritional status. To assess whether the trajectory of RDT positivity over time differed between nutritional status, we used a mixed-effects logistic regression model.

The rate of RDT positivity rate during the monthly home visits was almost similar in the two groups of children. The RDT positivity rate increased overtime during SMC period, regardless of the nutritional status of children. RDT positivity in October and November was slightly higher in malnourished children than in those with an ANS but the overall difference was not statistically significant, indicating no effect of nutritional status on the occurrence of asymptomatic infections. Fig 2 shows the RDT positivity rate by nutritional status over the study period.

## Discussion

In this study, there were more malnourished children than those with an adequate nutritional profile at the time of the SMC delivery in the health district of Nanoro. This could be explained by the coincidence of SMC period with the period of food shortage with insufficient children’s daily rations quantitatively and qualitatively in the villages. Meals have then low content in essential nutrients such as proteins, vitamins and minerals, leading to a depletion of the body’s energy reserves and plunging children into malnutrition. A trend towards an increase in the prevalence of undernutrition, specifically in July and November, the periods corresponding to the SMC, has also been reported in the region [31].

The high incidence of uncomplicated malaria in malnourished children could be explained by several factors. First,it may be due to a deficit in the immune system’s defence mechanisms in undernourished children compare to those with ANS. This hypothesis seems more plausible in view of asymptomatic infections which were almost similar between the two groups of children (similarly exposed to mosquitoes) suggesting that nutritional status has no impact on the onset of malaria infection, but rather contribute to prevent asymptomatic cases from progressing to uncomplicated or severe clinical forms [32,33]. Indeed undernourished children may have a reduced capacity to produce an adequate antimalarial immune response and react less effectively to the infection of pathogens, which normally triggers the production of specific antibodies directed against these pathogens. Thus, in malnourished children, the specific antimalarial antibodies produced to control infections may be relatively low compared with those of children with ANS [34,35].For example, a low prevalence of antimalarial immune responders and specific IgG antibodies in malnourished children, compared to those with an ANS was previously reported [36]. This could result from micronutrients deficiencies such as iron, zinc and vitamin A, which are common in malnourished children, and have a direct impact on the immune system strength to react to infectious disease [15].Several studies have shown that these deficiencies contribute significantly to malaria-related morbidity and mortality [37,38]. In such a context, supplementation with these micronutrients could help to reduce the incidence of infections including malaria, through strengthening children’s immune systems [25,^39^-^40^–].

Besides the difference in immune response, this increase malaria incidence in undernourished children could also be attributable to poor absorption of SMC drug. Such inadequate absorption could lead to bioavailability problems, and consequently reduced exposure to the drugs (underdosing), affecting their potential efficacy [41,42]**.**Several studies evaluating the pharmacokinetics of antimalarial treatments drugs in malnourished children compared to those with ANS and reported that children suffering from severe acute malnutrition were at higher risk of under-exposure to antimalarial drugs, as well as an increased risk of re-infection, compared to children with ANS [20,43]. Other studies have also reported that moderately malnourished children may be at similar risk of under-exposure to antimalarials [42,44].

The risk of clinical malaria was higher in undernourished children than in those with an ANS. Several studies have reported the same trend, establishing an association between undernutrition and clinical malaria in children under 5 years of age [38,45–47].However, Gebreegziabher et al. observed no significant association, while noting a higher number of malaria infections in children with a lower MUAC, HAZ and WAZ [48]. Other authors have also highlighted the high prevalence of malaria among malnourished school-age children although no formal association was established [49,50]. Moreover, the link between malnutrition and the effectiveness of antimalarial treatments has been reported, with undernutrition being correlated with a higher risk of recrudescence [43,51,52]. All these results suggest that undernutrition has a negative influence on malaria-related morbidity and mortality, and therefore it is crucial to take this aspect into account in all malaria control interventions.

In terms of age, children aged two years and over had a higher risk to present an uncomplicated malaria than those under two years. This effect could be attributed to the nutritional deficiencies associated with weaning children at this age. In fact, the study area, children aged two and over are generally weaned at this age and receive fewer food supplements to compensate for breast milk, which is particularly rich in nutrients [53,54]. On the other hand, children under the age of two still benefit from breastfeeding, which protects them from nutritional deficiencies and potential infections [55]. Indeed, the average duration of breastfeeding in Burkina Faso is 24.5 months, and longer in rural areas, 26.3 months [56]. Furthermore, increased exposure to vectors could explain the higher risk of malaria in children over the age of 2 as they. are generally more independent and spend more time outdoors, which increases their contact with the mosquitoes that cause malaria [57].

This study did not differentiate the type of malnutrition due to limited sample size and this represents a limitation of this study as the observed forms of undernutrition (stunting, wasting and underweight) could potentially differ in malaria susceptibility and antimalarial pharmacokinetics. Indeed previous studies reported differences in the risk of malaria infection according to malnutrition type [47,50,58,59]. For instance, wasting was highly associated with the risk of infections than stunting and underweight [47]. Furthermore, this study did not capture several important confounders, including household use of insecticide-treated nets, socioeconomic status, parental education, and proximity to health facilities. These factors may influence both nutritional status and malaria risk, and their absence represents a limitation. In addition, the absence of detailed dietary intake such as Food Frequency Questionnaire or 24-hour dietary recall precluded a comprehensive assessment of dietary patterns, the intake of macro- and micronutrients relevant to infectious processes, nutritional status and food access. Future studies should incorporate such variables to better elucidate the complex relationship between nutritional status and malaria.

From a public health policy perspective, a strategy involving micronutrient supplementation for malnourished children during the SMC period could be considered. This approach could build on existing programs, such as SMC +, which aims to integrate or combine several complementary interventions with the SMC campaign. Among the strategies currently incorporated are acute malnutrition screening, identification of unvaccinated or under-vaccinated children, community-level diagnosis and treatment of malaria by community health workers, as well as the identification and destruction of mosquito breeding sites. In this context, targeted micronutrient supplementation particularly formulations with low iron content could also be integrated into these interventions.

## Conclusion

Our study showed that nutritional status of children under SMC coverage influenced significantly the impact of SMC intervention. There was an increased risk of malaria infection in undernourished children compared to those with an adequate nutritional status suggesting the need of combining SMC intervention with nutrients supplementation to achieve best impact for malaria control in food insecure areas.

## Supporting information

S1 Data Data set for Malaria and malnutrition in children under the Seasonal Malaria Chemoprevention (SMC) coverage in the health district of Nanoro, Burkina Faso. (XLS)

## Abbreviations

ANS: Adequate nutritional status
AQSP: Amodiaquine +Sulfadoxine-Pyrimethamine
CRUN: Clinical Research Unit of Nanoro
HDSS: Health and Demographic Surveillance System
RDT: Rapid Diagnosis
SMC: Seasonal Malaria Chemoprevention
